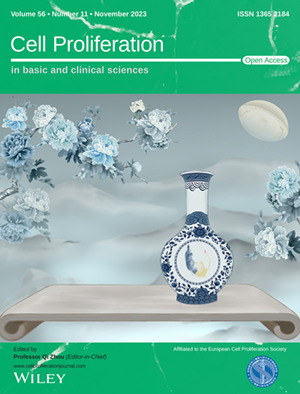# Featured Cover

**DOI:** 10.1111/cpr.13574

**Published:** 2023-11-03

**Authors:** Meng‐Chao Zhu, Wei Hu, Lei Lin, Qing‐Wen Yang, Lu Zhang, Jia‐Lin Xu, Yi‐Tong Xu, Jia‐Sheng Liu, Meng‐Di Zhang, Xiao‐Yu Tong, Kai‐Yi Zhu, Ke Feng, Yi Feng, Jian‐Zhong Su, Xiu‐Feng Huang, Jin Li

## Abstract

The cover image is based on the Original Article *Single‐cell RNA sequencing reveals new subtypes of lens superficial tissue in humans* by Meng‐Chao Zhu et al., https://doi.org/10.1111/cpr.13477.